# Radiosensitivity-Related Genes and Clinical Characteristics of Nasopharyngeal Carcinoma

**DOI:** 10.1155/2020/1705867

**Published:** 2020-11-22

**Authors:** Yongmei Dai, Yue Zhang, Mi Yang, Liang Zhou, Hua Pan, Ting Xiao, Lu Yuan, Yuting Wu, Min Chen, Longhua Chen, Jian Guan

**Affiliations:** ^1^Department of Oncology, Shengli Clinical Medical College of Fujian Medical University, Fujian Provincial Hospital, Fujian 350004, China; ^2^Department of Radiation Oncology, Nanfang Hospital, Southern Medical University, Guangzhou, Guangdong 510515, China; ^3^Department of Radiation Oncology, First Affiliated Hospital of Zhengzhou University, Zhengzhou 450000, China; ^4^Guangdong Provincial Key Laboratory of Tropical Disease Research, Department of Toxicology, School of Public Health, Southern Medical University, Guangzhou 510515, China

## Abstract

**Materials and Methods:**

Clinicopathological data of 185 patients with NPC treated at Nanfang Hospital of Southern Medical University between January 2013 and December 2014 were retrospectively analyzed. SPSS statistical software was used to analyze the clinicopathological data related to radiotherapy efficacy. Three patients who achieved complete remission and three with disease progression after CRT were selected. Differentially expressed genes (DEGs) were screened via mRNA microarray analysis of primary diagnostic endoscopy specimens.

**Results:**

The peripheral blood leukocyte count, platelet count, and EBV-DNA copy number in NPC patients who were resistant to radiotherapy were higher than those in NPC patients who were sensitive to radiotherapy. The RobustRankAggreg (RRA) analysis method identified 392 DEGs, and the 66 most closely related genes among the DEGs were identified from the PPI network.

**Conclusion:**

The results of this study indicate that screening for DEGs and pathways in NPC using integrated in silico analyses can help identify a series of genetic and clinical signatures for NPC patients treated with neoadjuvant chemotherapy followed by concurrent chemoradiotherapy.

## 1. Introduction

Nasopharyngeal carcinoma (NPC) is a highly malignant tumor originating from the epithelium; this disease shows a specific ethnic and geographical distribution [1]. NPC is prevalent among South and East Asian populations [2]. Lymph node and distant metastasis can occur early during disease progression without any obvious symptoms. More than 70% of NPC patients have locally advanced disease upon confirmation of their diagnosis. Improvements in treatment methods and implementation of comprehensive treatment strategies have substantially increased the 5-year survival rate of early-stage NPC patients to approximately 85% at present [3, 4]. Radiotherapy is an essential component of the treatment of nondisseminated disease with curative intent, and its application in conjunction with chemotherapy prolongs survival [5]. Because NPC is sensitive to radiotherapy and the nasopharynx has a unique anatomical position, the comprehensive treatment of NPC is mainly radical radiotherapy [6]. Although local control of NPC has improved significantly due to advances in radiotherapy and comprehensive treatments, some patients do not benefit from radiotherapy due to the radiation resistance caused by local recurrence and distant metastasis [7]. Moreover, a poor response to chemoradiotherapy is related to substantial adverse effects and high medical costs.

In the era of personalized medicine, individualized radiotherapy guided by biomarkers and/or combination therapy has begun to emerge [8]. Many studies have evaluated whether incorporating other clinical factors and molecular biomarkers into the current anatomical staging system can better predict survival because this system is insufficient in predicting the prognosis or therapeutic effect [9]. This type of research mainly focuses on the specific genetic characteristics relating to the diagnosis, prognosis, or prediction of treatment response [10]. Therefore, establishing a model to predict the survival outcome of patients with locally advanced NPC will help identify the patients who would benefit most from intensity-modulated radiation therapy (IMRT). The aim of this study is to explore the relevant clinical factors or sensitivity predictors of chemoradiotherapy, which will help guide the selection of individualized treatment options for NPC patients, improve the curative effect, and avoid ineffective or excessive treatment.

## 2. Methods

### 2.1. Patient and Specimen Selection

#### 2.1.1. Patient Selection

Clinicopathological data of 185 patients with NPC from Nanfang Hospital of Southern Medical University between January 2013 and December 2014 were retrospectively analyzed. The inclusion criteria were as follows: (1) biopsy-proven NPC, (2) initial treatment with no previous antitumor therapies, and (3) stage I to IVb NPC according to the 2010 edition of the American Joint Committee on Cancer (AJCC) staging system. Other eligibility criteria included a Karnofsky score > 70, age 18–70 years old, and normal electrocardiography (ECG), complete blood count, and liver and renal function results. The baseline examination included nasopharynx and neck MRI, chest and upper abdomen enhanced CT, and whole-body bone scanning. The protocol was approved by the ethics committee of Nanfang Hospital NFEC-2018-013 and implemented in accordance with the principles of the Declaration of Helsinki. All participants provided informed consent.

#### 2.1.2. Specimen Selection

Three patients with complete remission and three with disease progression after CRT were selected (Table 1). The principle of specimen selection was as follows. (1) Resistant group: three months after the end of radiotherapy and chemotherapy, no obvious regression of the tumor was confirmed by imaging, and the pathology confirmed tumor residue. (2) Sensitive group: three months after the end of radiotherapy and chemotherapy, the tumor responded completely or partially to the treatment as confirmed by imaging and pathology. The endoscopic specimens obtained from the first diagnosis were used for the mRNA microarray.

#### 2.1.3. Therapeutic Method

(1) Chemotherapy: stage I patients received concurrent radiotherapy and chemotherapy while stage II-IVb patients received induction chemotherapy (IC) and concurrent chemotherapy. IC consisted of paclitaxel+fluorouracil (TP) or cisplatin+fluorouracil (PF) regimens. Two cycles of chemotherapy were repeated every three weeks, followed by concurrent radiotherapy (RT) and cisplatin administration (40 mg/m^2^) weekly on weeks 7 through 14. (2) Radiotherapy: IMRT was used in radical radiotherapy, and the target area was defined according to the ICRU [11] 71 and 83 [12] recommendations and the international consensus guidelines [13]. Reductions in nasopharyngeal and neck masses were examined by MRI or CT three months after treatment. RECIST 1.1 criteria were used to evaluate treatment efficacy [14]. A complete response (CR) was defined by the disappearance of all lesions, with nodes measuring <10 mm and the EBV-DNA copy number reduced to a normal level. An increase of ≥20% from the nadir or baseline was defined as progressive disease (PD). A decrease of at least 30% in the sum of diameters of target lesions in relation to the baseline sum diameters was defined as partial response (PR). Stable disease (SD) was defined as any result between PR and PD. Patients with either CR or PR were classified as the sensitive group while those with SD and PD were classified as the resistant group. The nasopharyngeal biopsy is required to confirm the residual in SD and PD patients

### 2.2. Customized mRNA Microarrays

The gene expression profile was obtained by an Affymetrix Human U133 Plus 2.0 chip. Total RNA was extracted and purified using RecoverAllTM Total Nucleic Acid Isolation (Cat. #AM1975, Ambion, Austin, TX, US) following the manufacturer's instructions. The initial sample of the chip experiment was the total RNA. Total RNA was examined on a NanoDrop ND-2000 spectrophotometer and an Agilent Bioanalyzer 2100 (Agilent Technologies, Santa Clara, CA, US). The qualified RNA could be used for subsequent chip experiments. No RNA degradation or DNA mixing occurred when at least two distinct bands representing 28S and 18S ribosomal RNA were available on the electrophoretogram. At least 2 micrograms of the sample is usually needed before purification. The total RNA was amplified, labeled, and purified using an Ovation FFPE WTA System (Cat. #3403, NuGEN, San Carlos, CA, US) and FL-Ovation™ cDNA Biotin Module V2 (Cat. #4200, NuGEN) following the manufacturer's instructions to obtain biotin-labeled cRNA. Array hybridization and washing were performed using a GeneChip® Hybridization, Wash and Stain Kit (Cat. #900720, Affymetrix, Santa Clara, CA, US) in a Hybridization Oven 645 (Cat. #00-0331-220V, Affymetrix) and a Fluidics Station 450 (Cat. #00-0079, Affymetrix) following the manufacturer's instructions. The slides were scanned by a GeneChip® Scanner 3000 (Cat. #00-00212, Affymetrix) and Command Console Software 4.0 (Affymetrix) with default settings. Raw data were normalized by the MAS 5.0 algorithm, Gene Spring Software 12.6.1 (Agilent Technologies, Santa Clara, CA, US).

### 2.3. mRNA Microarray Sorting and Analysis

The linear microarray data model (limma) package [15] in R language was used to screen the differentially expressed genes (DEGs) between the radiosensitive group and the resistant group. Differentially expressed genes (DEGs) were screened according to the fold change (FC) compared with the control values, and those with FC ≥ 2 and *p* value <0.05 according to the *t*-test were considered DEGs. Then, GO analysis and KEGG enrichment pathway analysis were performed on the obtained differential genes. Details about the in silico analyses are provided in the supplementary information (available here).

#### 2.3.1. Statistical treatment

The SPSS 22.0 statistical software was used to analyze the continuous variables as the mean ± standarddeviation. The statistics are expressed as percentages, and the *χ*^2^ test and *t*-test were used for significance tests. A *p* value of <0.05 was considered statistically significant.

## 3. Results

### 3.1. Characteristics of Clinical Data

The sensitive group included 124 patients with an average age of 46.1 years, and the resistant group included 61 patients with an average age of 46.1 years. No significant differences in gender ratio, age distribution, smoking history, or drinking history between the two groups were found. The T staging and N staging of the resistant group were significantly higher than those of the sensitive group (*p* = 0.001, 0.005, respectively). No significant difference was found in the probability of metastasis between the two groups before treatment (*p* = 0.577). The results showed that the proportion of differentiated tumors in the resistant group was significantly higher than that in the sensitive group (*p* = 0.01) (Table 2).

### 3.2. Analysis of Hematological Examination and Treatment Plan before Chemoradiotherapy

The level of EBV DNA in the resistant group was significantly higher than that in the sensitive group (resistant group vs. sensitive group = 14.24 ± 35.86 × 104copies/mlvs. 2.08 ± 8.04 × 104copies/ml, *p* = 0.028). A total of 132 patients received induction chemotherapy: 87 in the sensitive group and 45 in the resistant group. No noticeable difference was observed in the proportion of patients receiving induction chemotherapy between the two groups. A total of 104 patients in the sensitive group received concurrent chemotherapy during radiotherapy, as did 41 patients in the resistant group. No significant difference was found in the cumulative dose of cisplatin between the two groups (sensitive group 138.62 ± 98.533mg, resistant group 113.28 ± 105.13mg, *p* = 0.110), and no significant difference was found in the number of patients with a cumulative dose of cisplatin > 200mg/m^2^ between the two groups. A total of 107 patients received adjuvant chemotherapy, and no significant difference was found in the proportion of adjuvant chemotherapy and the cumulative dose of cisplatin between the two groups.

All patients were assessed for nasopharyngeal lesion regression within 1 year after treatment. The response rate of radiotherapy and chemotherapy in the sensitive group was 98.4% (122/124). In the resistant group, 49.2% (30/61) of the lesions shrunk after the treatment. Among them, 83.3% (25/30) of the patients had an in situ recurrence of nasopharyngeal tumors within 2 years, and 13.3% (4/30) had an in situ recurrence of nasopharyngeal tumors within 3 years. Two patients were reexamined in the external hospital after treatment, and the case data of the other hospital indicated that the nasopharyngeal tumor had recurred. However, the imaging data after treatment could not be provided, and the curative effect was not evaluated. Twenty-eight patients experienced distant metastasis during observation, including 13 cases of metastasis in the sensitive group and 15 cases in the resistant group. The statistical analysis showed that the proportion of distant metastases was higher in the resistant group than in the sensitive group, and this difference was significant (*p* = 0.016) (Table 3).

### 3.3. Results of the Gene Chip Data Analysis

#### 3.3.1. DEGs between Two Groups

**Figure 1:**
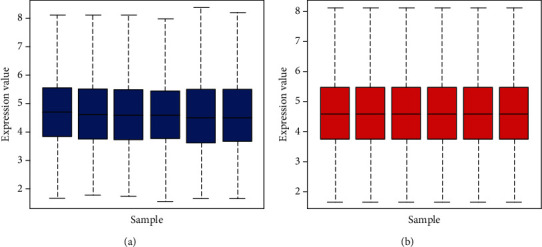
Standardization of gene expression. The blue bar represents the data before normalization, and the red bar represents the normalized data. The abscissa represents each sample, and the ordinate represents the quantity of expression.

**Figure 2:**
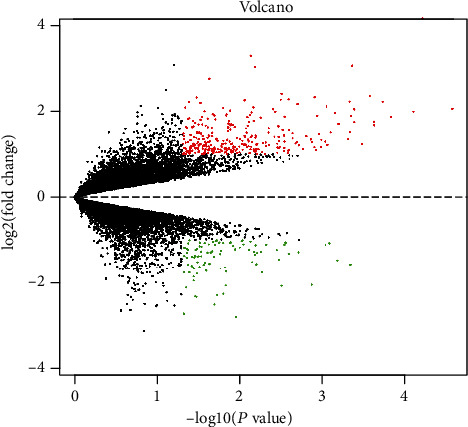
DEGs between two sets of samples. The upregulated genes (red dots) were selected based on FC > 2.0 and a corrected *p* value of <0.05. The downregulated genes (green dots) were screened based on an FC ≤ −2.0 and a corrected *p* value of <0.05. Genes with no significant difference in the expression are indicated by the black spot. Abbreviation: FC: fold change.

**Figure 3:**
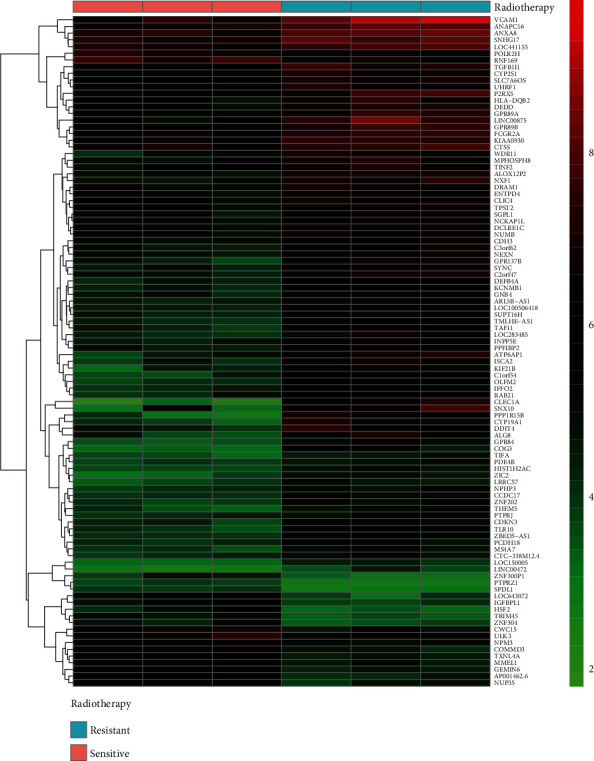
Hierarchical clustering heat map of DEGs screened based on a ∣foldchange | >2.0 and a corrected *p* value <0.05. The upregulated genes (red) were screened based on an FC > 2.0 and a corrected *p* value of <0.05. The downregulated genes (green) were screened based on an FC ≤ −2.0 and a corrected *p* value of <0.05. Genes with no significant difference in the expression are indicated by black boxes. Gray indicates that the signal intensity of the gene was not sufficiently high to be detected. Abbreviation: FC: fold change.

The expression microarray results are listed in Figure 1. The microarray results identified 392 differentially expressed genes between the two groups: 92 downregulated genes and 300 upregulated genes.  Figure 2 shows the differential expression of multiple genes between the two groups included in the microarray.  Figure 3 shows the cluster heat map of the top 200 DEGs.

#### 3.3.2. GO Term Enrichment Analysis of DEGs

**Figure 4:**
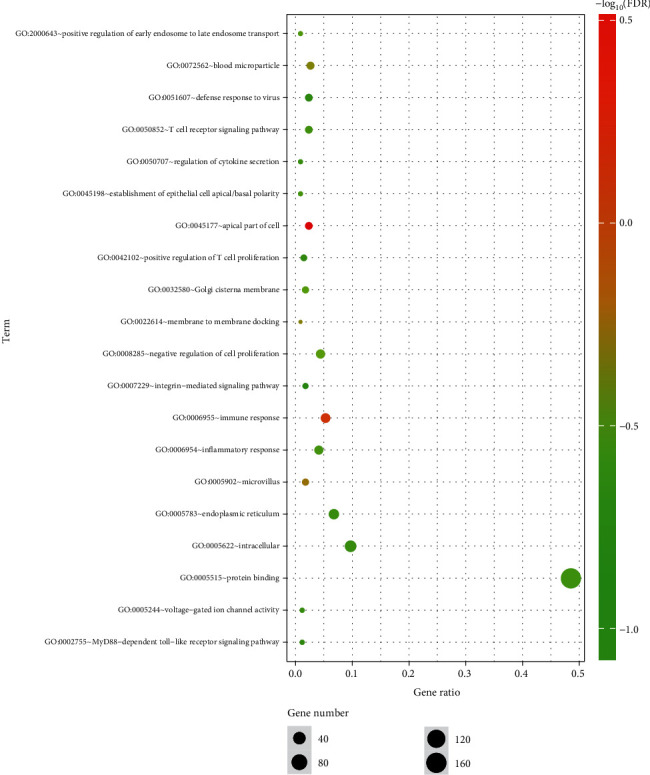
GO enrichment analysis of DEGs. Notes: The number of genes (“count”) divided by the number of total genes is the gene ratio. The size of the dots represents the number of core genes, and the color indicates the adjusted *p*. Only pathways with an adjusted *p* < 0.05 were enriched.

The results of the gene ontology analysis of DEGs include three parts: biological processes, molecular functions, and cellular components (Figure 4).  Table 4 shows the results of the gene ontology enrichment analysis of DEGs in NPC. In the biological process group, the DEGs were mainly concentrated in pathways relating to the immune response, blood microparticles, negative regulation of cell proliferation, positive regulation of early endosome to late endosome transport, establishment of epithelial cell apical/basal polarity, inflammatory response, T cell receptor signaling pathway, regulation of cytokine secretion, MyD88-dependent Toll-like receptor signaling pathway, positive regulation of T cell proliferation, defense response to virus, integrin-mediated signaling pathway, regulation of cell shape, protein localization to organelle, detection of triacyl bacterial lipopeptide, positive regulation of innate immune response, regulation of cytoskeleton organization, platelet activation, regulation of cell size, intracellular pH reduction, Toll-like receptor TLR1:TLR2 signaling pathway, cellular response to triacyl bacterial lipopeptide, and positive regulation of type I interferon production. In the molecular function group, the DEGs were mainly enriched in protein binding and voltage-gated ion channel activity. In the cell composition group, the DEGs were mainly enriched in the apical part of the cell, microvillus, membrane-to-membrane docking, Golgi cisterna membrane, endoplasmic reticulum, intracellular environment, membrane, endocytic vesicle lumen, filopodium, filopodium assembly, nucleoplasm, Toll-like receptor 1-Toll-like receptor 2 protein complex, microvillus membrane, extracellular matrix organization, ruffle membrane, Golgi-associated vesicle membrane, and TCR signalosome. These results show that most DEGs are significantly enriched in protein binding, the nucleoplasm, the membrane, the intracellular environment, and the endoplasmic reticulum.

#### 3.3.3. KEGG Pathway Analysis of DEGs

**Figure 5:**
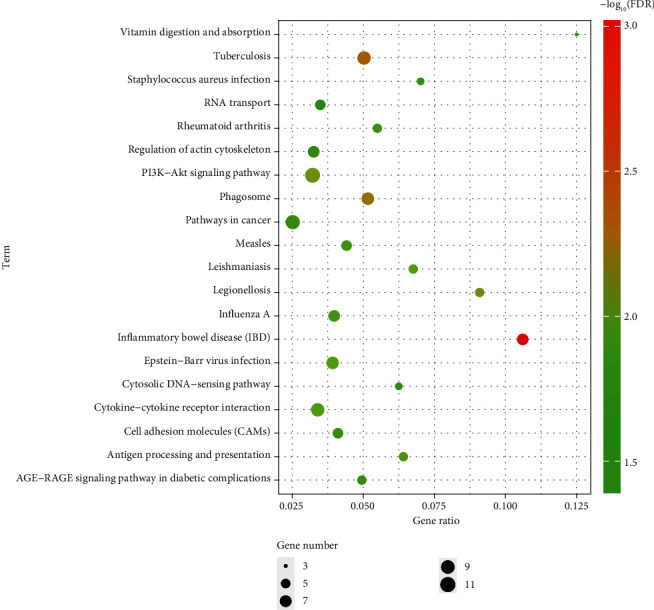
KEGG pathway analysis of DEGs in NPC. Notes: The number of genes (“count”) divided by the number of total genes is the gene ratio. The size of the dots represents the number of core genes, and the color indicates the adjusted *p* value. Only pathways with an adjusted *p* < 0.05 were enriched.

The DEGs obtained in the microarray were analyzed by the online analysis database KOBAS 3.0 (http://kobas.cbi.pku.edu.cn/).  Table 5 and Figure 5 show the most significant enrichments of DEGs from the KEGG analysis. The signaling pathways of DEGs were mainly enriched in the PI3K-Akt signaling pathway, tuberculosis, Epstein-Barr virus infection, phagosomes, cytokine-cytokine receptor interactions, inflammatory bowel disease (IBD), legionellosis, influenza A, leishmaniasis, and antigen processing and presentation.

#### 3.3.4. Analysis of DEGs in NPC Using a PPI Network

**Figure 6:**
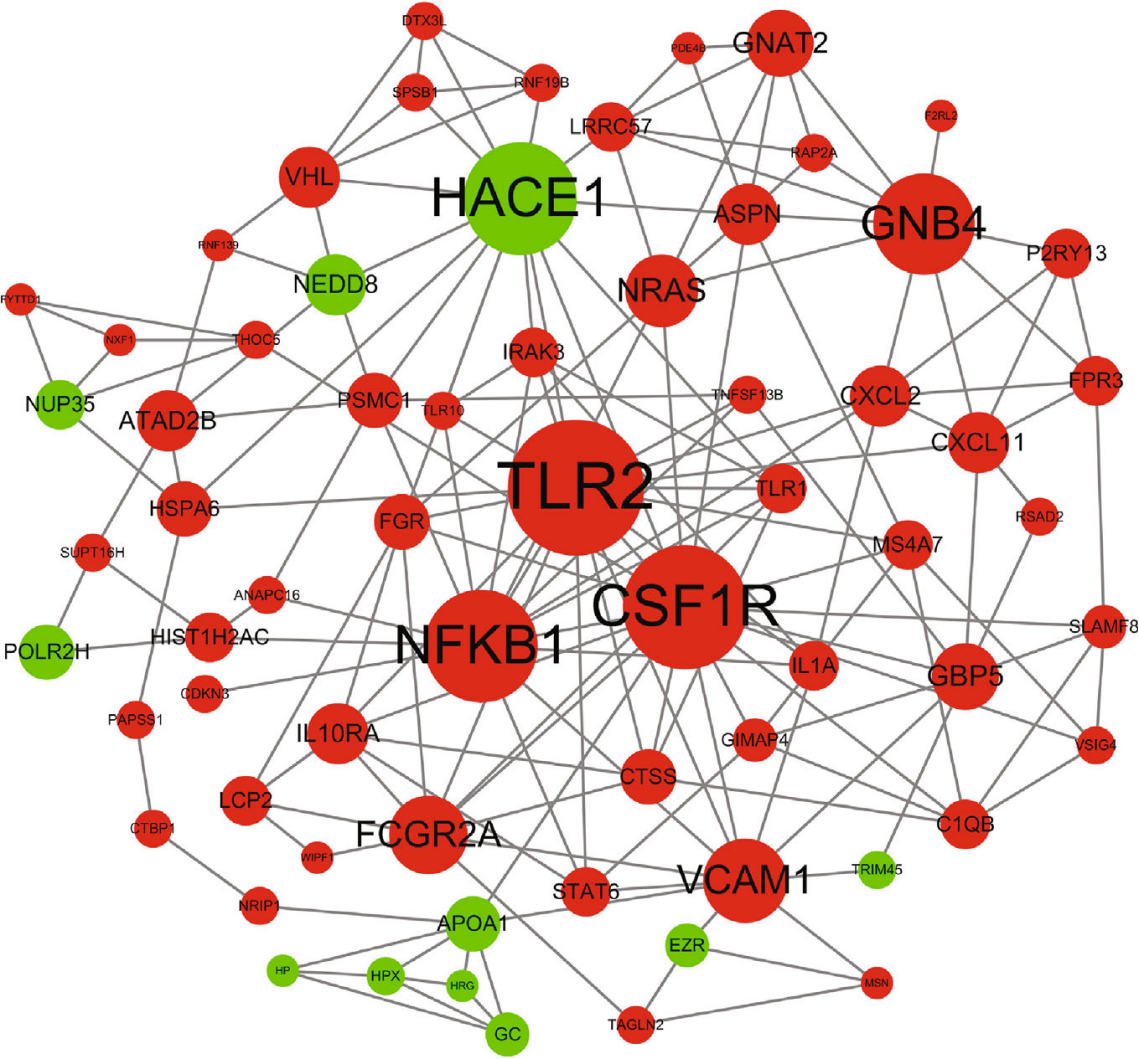
PPI network. Notes: The more proteins interact with each other, the larger the dot is, indicating that the more central the network is, the more critical and important the role is. Red indicates upregulated genes, and green indicates downregulated genes. Abbreviation: PPI: protein–protein interaction.

Using the STRING database [16] (http://string-db.org) to construct a protein-protein interaction (PPI) network, we obtained 392 DEGs, including 300 upregulated genes and 92 downregulated genes. After pruning away the orphaned and loosely connected nodes, an interactome network of DEGs was constructed, as shown in Figure 6. The 66 hub genes, including 55 upregulated genes and 11 downregulated genes, showing the most significant interaction are listed in Table 6.

## 4. Discussion

NPC is a malignant tumor that is very sensitive to radiation, and its sensitivity can differ depending upon the degree of tumor differentiation. A lower degree of differentiation indicates a higher sensitivity to radioactivity. In China, especially in south China, the most common pathological type is the nonkeratinized undifferentiated type (WHO type II) [17], which is very sensitive to radiation. However, many studies have shown that radiotherapy can not only kill the tumors but also change the expression level of many genes and proteins [18, 19]. These changes can reduce the sensitivity of the tumor to radiation and thus lead to radiation resistance. Radiotherapy resistance is the main cause of failure in the treatment of nasopharyngeal carcinoma [20]. Therefore, exploring the molecular mechanism of radiotherapy resistance of nasopharyngeal carcinoma is very important in improving the effects of radiotherapy and improving the prognosis of patients with nasopharyngeal carcinoma.

Among the 185 patients included, 61 patients with NPC had poor curative effects or local recurrence in the short term. Comparing the differentiation of tumors in the two groups, the proportion of differentiation types in the resistant group was higher than that in the sensitive group (14.3% vs. 3.3% in the resistant group, *p* = 0.01). This result again suggests that more differentiated tumors are less sensitive to radiotherapy. Epstein-Barr virus infection is very common in NPC patients. Mutirangura [21] in 1997 and LO [22] in 1999 found that the positive rate of EBV-DNA and the copy number were significantly higher in the sera of NPC patients than in the sera of healthy controls. Subsequent studies confirmed that the positive rate and level of EBV-DNA detection in nasopharyngeal cancer patients with recurrence or metastasis (the median quantitative concentration of EBV in the recurrence or metastasis group was 32350 copies/ml) were significantly higher than those in patients who achieved clinical remission. In the sensitive group, the median concentration of EBV-DNA was 0 copies/ml (*p* = 0.01) [23]. During follow-up, the researchers found that plasma EBV-DNA levels were elevated in patients with recurrent nasopharyngeal cancer approximately six months before the onset of clinical symptoms. Similar to our results, the EBV level in the blood of the nasopharyngeal-cancer-resistant group was significantly higher than that of the sensitive group: the median concentration of EBV in the resistant group was 142,400 copies/ml compared with 20,800 copies/ml in the sensitive group (*p* = 0.028). Therefore, we speculated on whether EBV infection and the EBV-DNA copy number are associated with radiosensitivity in NPC.

According to the current theory regarding the molecular biology of cancer, the radiosensitivity of cancer cells may be regulated by a complex network. Differences in any link in such a system, such as a mutation or a difference in expression in a single gene, may affect radiosensitivity. At present, although the research on radiosensitivity has been extensive, most studies have examined only one or a limited number of genes and their expression products. To further understand the molecular mechanism of radiosensitivity of cancer cells, simultaneously detecting the expression of several genes related to this network is necessary. Gene chip technology provides a semiquantitative analysis of a large number of genes at the whole-genome level. It can compare differences in gene expression among different samples at the same time, reveal new genes, and analyze gene interaction networks through a clustering analysis and functional enrichment. Therefore, gene chip technology is widely used in research on a variety of cancers, such as cholangiocarcinoma [24], colorectal cancer [25], breast cancer [26], and pancreatic cancer [27]. However, the identification of DEGs and hub genes will help us better understand the molecular mechanism of NPC progression and consequently develop more biomarkers, which will be helpful for the study of the early diagnosis and treatment of NPC [28, 29]. However, the gene research in NPC mainly focuses on the occurrence, development, recurrence, and metastasis of NPC [30, 31]. Few similar studies on radiation resistance have been published.

In this study, we used the gene expression profile technology of an mRNA microarray to obtain a large number of DEGs and then used molecular biology information technology for data processing to identify molecular markers that can predict the efficacy of CRT. The results identified 392 DEGs, including 300 upregulated genes and 92 downregulated genes. The DEGs in NPC were analyzed by GO functional annotation, which showed that most DEGs are significantly enriched in protein binding, the nucleoplasm, membranes, intracellular environment, and the endoplasmic reticulum. Thus, the differential genes may be mainly related to cells. At present, many studies have shown that the radiosensitivity is closely related to the cell cycle [32].

The signaling pathways of DEGs determined by the KEGG signal pathway analysis were mainly enriched in the PI3K-Akt signaling pathway, tuberculosis, Epstein-Barr virus infection, phagosomes, cytokine-cytokine receptor interactions, inflammatory bowel disease (IBD), legionellosis, influenza A, leishmaniasis, and antigen processing and presentation. The PI3K-Akt signaling pathway is closely related to tumorigenesis and cancer progression. Many studies have been published on the relationship between the PI3K-Akt signaling pathway and nasopharyngeal carcinoma. They include, for example, how microRNA-29 targets FGF2 and inhibits the proliferation, migration, and invasion of nasopharyngeal carcinoma cells via the PI3K/AKT signaling pathway; CHL1 suppresses tumor growth and metastasis in nasopharyngeal carcinoma by repressing the PI3K/AKT signaling pathway via interaction with Integrin *β*1 and Merlin [33]; and microRNA-29 targets FGF2 and inhibits the proliferation, migration, and invasion of nasopharyngeal carcinoma cells via the PI3K/AKT signaling pathway [34]. Therefore, the PI3K/Akt signaling pathway plays an important role in the occurrence, development, recurrence, and metastasis of nasopharyngeal carcinoma. In this study, we found that the differentially expressed genes were mainly enriched in the PI3K Akt signaling pathway, warranting further study.

Using the STRING database to construct a PPI network, we obtained 66 hub genes, including 55 upregulated genes and 11 downregulated genes. The mechanisms and functions of these genes and their roles in the radiotherapy resistance of NPC should be further studied.

For a long time, research on improving the effect of radiotherapy has been focused on cancer cells; however, in recent years, research has focused on the tumor microenvironment (TME). As a result, most of the classical radiobiology dogma fails to consider the effect of radiotherapy on the TME, and the reaction of radiotherapy to the TME may be very important for the success or failure of the treatment. Therefore, the attempt to combine radiotherapy with new biological targeted therapy is usually based on the potential to enhance cancer cell death induced by radiotherapy rather than on the potential to enhance radiosensitization by influencing TME. Many strategies have been proposed to overcome the radiation resistance of tumor cells, but little research has been conducted on the TME-mediated mechanisms of radiation resistance and how to circumvent these mechanisms [35]. In recent years, radiation therapy and the immune microenvironment have become popular research topics [36, 37]. In this study, the results of the gene chip analysis showed that the genes differentially sensitive and insensitive to CRT in NPC were associated with the immune microenvironment, including the immune response, T cell receptor signaling pathway, positive regulation of T cell proliferation, positive regulation of the innate immune response, Toll-like receptor TLR1:TLR2 signaling pathway, TCR signalosomes, and intracellular pH reduction. However, how these different genes affect the efficacy of preoperative chemoradiotherapy is unclear; further basic scientific and clinical research studies are needed.

## 5. Conclusion

The peripheral blood leukocyte count, platelet count, and EBV-DNA copy number were higher in patients with NPC who were resistant to radiotherapy than in those who were sensitive to radiotherapy. The results of this study show that bioinformatics analyses of gene chip data can help us to identify and screen a series of gene characteristics related to NPC sensitivity to radiotherapy and chemotherapy. The genes involved in the mechanism of radiosensitivity in NPC patients are closely related to platelet aggregation, inflammatory factors, and EBV infection pathways, which will be of great clinical value in the future. Monitoring and controlling the cytokines related to inflammation and immunity can prevent and delay the occurrence and development of tumors to a certain extent. Additional follow-up studies should explore the relevant factors or predictive indicators of chemoradiotherapy sensitivity, which will help guide the selection of individualized treatment options for NPC patients, improve the curative effect, and avoid ineffective or excessive treatment.

Some limitations to this study exist. We analyzed a series of differential genes of radiotherapy resistance in nasopharyngeal carcinoma; however, due to time and financial constraints, we did not conduct subsequent functional verification. Further molecular biological experiments are required to confirm the function of the identified genes associated with NPC.

## Figures and Tables

**Table 1:** Patient information for mRNA microarrays.

Sample	Gender	Age	T stage	N stage	M stage	TNM stage
1^∗^	Male	61	1	1	0	III
2^∗^	Female	28	4	1	0	IVa
3^∗^	Female	34	3	2	0	IVa
4^∗∗^	Female	48	2	2	0	IVa
5^∗∗^	Male	37	2	1	0	III
6^∗∗^	Female	34	4	1	0	IVa

^∗^sensitive group; ^∗∗^ resistant group.

**Table 2:** Basic clinical data.

Items	Sensitive group	Resistant group	*p* value
Sex			
Male	87 (70.2%)	49 (80.3%)	0.16
Female	37 (29.8%)	12 (19.7%)	
Age	46.1 ± 11.5	46.1 ± 10.8	0.98
Smoking	47 (37.9%)	29 (47.5%)	0.27
Drinking	11 (8.9%)	8 (13.1%)	0.44
Family history	23 (18.5%)	13 (21.3%)	0.695
Tumor differentiation			
Differentiated	4 (3.3%)	8 (14.3%)	0.01
Undifferentiated	119 (96.7%)	48 (85.7%)	
T staging			
T1	16 (12.9%)	3 (4.9%)	0.001
T2	25 (20.2%)	6 (9.8%)	
T3	45 (36.3%)	15 (24.6%)	
T4	38 (30.6%)	37 (60.7%)	
N staging			
N0	9 (7.3%)	11 (18.0%)	0.005
N1	34 (27.4%)	5 (8.2%)	
N2	76 (61.3%)	40 (65.6%)	
N3	5 (4.0%)	5 (8.2%)	
M staging			
Mx	4 (3.2%)	2 (3.3%)	0.577
M0	116 (93.5%)	55 (90.2%)	
M1	4 (3.2%)	4 (6.6%)	

**Table 3:** Adjuvant examinations and treatment options prior to chemoradiotherapy.

Items	Sensitive group	Resistant group	*p* value
Leukocyte count, G/L	6.95 ± 1.96	8.02 ± 2.35	0.01
Neutrophil count, G/L	4.41 ± 1.65	5.3 ± 1.99	0.01
Percentage of neutrophils, %	62.58 ± 8.95	65.62 ± 9.66	0.037
Platelet count, G/L	249.89 ± 68.96	281.25 ± 70.53	0.005
EBV antibody			
Positive	56 (60.9%)	20 (57.1%)	0.840
Negative	36 (39.1%)	15 (42.9%)	
EBV-DNA copy numbers 10^4^ copies/ml	2.08 ± 8.04	14.24 ± 35.86	0.028
Time from diagnosis to RT	17.09 ± 35.771	32.33 ± 34.942	0.011
Cumulative dose of cisplatin in concurrent chemotherapy	138.62 ± 98.533	113.28 ± 105.13	0.110
Cumulative dose of cisplatin in concurrent chemotherapy (>200 mg/m^2^)	31 (30.4%)	11 (21.2%)	0.255
Distant metastasis			
Yes	13 (10.5%)	15 (24.6%)	0.016
No	111 (89.5%)	46 (75.4%)	

**Table 4:** GO analysis of DEGs associated with NPC.

Term	Description	Count	*p* value
GO:0045177	Apical part of the cell	8	0.000206
GO:0006955	Immune response	18	0.000551
GO:0005902	Microvillus	6	0.002229
GO:0022614	Membrane-to-membrane docking	3	0.00255
GO:0072562	Blood microparticle	9	0.003304
GO:0008285	Negative regulation of cell proliferation	15	0.005509
GO:2000643	Positive regulation of early endosomal to late endosomal transport	3	0.006912
GO:0032580	Golgi cisterna membrane	6	0.007681
GO:0045198	Establishment of epithelial cell apical/basal polarity	3	0.008792
GO:0006954	Inflammatory response	14	0.009314
GO:0050852	T cell receptor signaling pathway	8	0.01067
GO:0050707	Regulation of cytokine secretion	3	0.013148
GO:0005515	Protein binding	165	0.013285
GO:0005783	Endoplasmic reticulum	23	0.015217
GO:0005622	Intracellular environment	33	0.015487
GO:0005244	Voltage-gated ion channel activity	4	0.015554
GO:0002755	MyD88-dependent Toll-like receptor signaling pathway	4	0.016183
GO:0042102	Positive regulation of T cell proliferation	5	0.016355
GO:0051607	Defense response to virus	8	0.018434
GO:0007229	Integrin-mediated signaling pathway	6	0.022738
GO:0016020	Membrane	48	0.026775
GO:0071682	Endocytic vesicle lumen	3	0.026816
GO:0008360	Regulation of cell shape	7	0.026966
GO:0033365	Protein localization to organelle	3	0.027197
GO:0030175	Filopodium	5	0.027746
GO:0046847	Filopodium assembly	3	0.030498
GO:0035354	Toll-like receptor 1-Toll-like receptor 2 protein complex	2	0.032006
GO:0005654	Nucleoplasm	58	0.032009
GO:0042495	Detection of triacyl bacterial lipopeptide	2	0.032252
GO:0045089	Positive regulation of innate immune response	3	0.033949
GO:0031528	Microvillus membrane	3	0.037025
GO:0051493	Regulation of cytoskeleton organization	3	0.037543
GO:0030168	Platelet activation	6	0.039723
GO:0008361	Regulation of cell size	3	0.041276
GO:0030198	Extracellular matrix organization	8	0.041455
GO:0032587	Ruffle membrane	5	0.043625
GO:0030660	Golgi-associated vesicle membrane	3	0.044525
GO:0036398	TCR signalosome	2	0.047624
GO:0051452	Intracellular pH reduction	2	0.047987
GO:0038123	Toll-like receptor TLR1:TLR2 signaling pathway	2	0.047987
GO:0071727	Cellular response to triacyl bacterial lipopeptide	2	0.047987
GO:0032481	Positive regulation of type I interferon production	4	0.049991

**Table 5:** KEGG pathway analysis of DEGs associated with NPC.

Pathway	ID	Gene count	*p* value	Corrected *p* value	Genes
Inflammatory bowel disease (IBD)	hsa05321	7	2.46E-06	0.000487	*STAT6*, *HLA-DQB1*, *IL21R*, *TLR2*, *IL2RG*, *NFKB1*, *IL1A*
Tuberculosis	hsa05152	9	2.85E-05	0.002826	*HLA-DQB1*, *IL10RA*, *ATP6AP1*, *TLR1*, *TLR2*, *NFKB1*, *FCGR2A*, *CTSS*, *IL1A*
Phagosome	hsa04145	8	6.66E-05	0.004396	*HLA-DQB1*, *ITGAV*, *ATP6AP1*, *TAP2*, *TLR2*, *FCGR2A*, *CTSS*, *THBS3*
Legionellosis	hsa05134	5	0.000138	0.00684	*CXCL2*, *PYCARD*, *HSPA6*, *TLR2*, *NFKB1*
PI3K-Akt signaling	hsa04151	11	0.000187	0.007419	*NRAS*, *ITGAV*, *TLR2*, *GNB4*, *IL2RG*, *NFKB1*, *THBS3*, *GHR*, *CSF1R*, *COL4A5*, *DDIT4*
Epstein-Barr virus infection	hsa05169	8	0.000402	0.012426	*POLR2H*, *POLR3F*, *FGR*, *IL10RA*, *PSMC1*, *HSPA6*, *NFKB1*, *HLA-DQB1*
Cytokine-cytokine receptor interaction	hsa04060	9	0.000488	0.012426	*TNFSF13B*, *IL10RA*, *IL21R*, *IL2RG*, *CXCL11*, *IL1A*, *GHR*, *CSF1R*, *CXCL2*
Leishmaniasis	hsa05140	5	0.000502	0.012426	*HLA-DQB1*, *TLR2*, *NFKB1*, *FCGR2A*, *IL1A*
Antigen processing and presentation	hsa04612	5	0.00063	0.013854	*HLA-DQB1*, *TAP2*, *HSPA6*, *CTSS*, *KLRC1*
Influenza A	hsa05164	7	0.000849	0.016811	*HLA-DQB1*, *PYCARD*, *HSPA6*, *RSAD2*, *NFKB1*, *NXF1*, *IL1A*

**Table 6:** Hub genes in NPC by PPI network.

Hub genes	Gene names
Upregulated genes	*ANApc16*, *MSN*, *FSMC1*, *ATAD2B*, *FYTTD1*, *TAGLN2*, *Tnfsf13B*, *TLR1*, *cdKN3*, *CTBp1*, *wipf1*, *NF-KB1*, *Spsb1*, *TLR2*, *NRAS*, *CXCL2*, *STAT6*, *VHL*, *LCP2*, *IL10RA*, *CTSS*, *HSPA6*, *FCGR2A*, *NRIp1 (RIP140)*, *THOC5*, *Il1A*, *RAp2A*, *RNF139*, *CXCL11*, *VSIG4*, *DTX3L (BBAP)*, *IRAK3 (IRAKM)*, *ms4a7*, *HIST1H2Ac*, *FGR*, *PDE4B*, *F2RL2*, *FPR3*, *RSAD2*, *SUPT16H*, *GIMAP4*, *RNF19B*, *LrRc57*, *p2RY13*, *SLAmF8*, *CIQB*, *CSFIR*, *GNAT2*, *VCAMI*, *ASPN*, *GNB4*, *GBP5*, *PApsS1*, *TLR10*, and *NXF1*
Downregulated genes	*HACE1*, *NEDD8*, *Nup35*, *POLR2H*, *HPX*, *HP*, *APOA1*, *HRG*, *GC*, *TRIM45*, *EZR*

Abbreviation: PPI: protein-protein interaction.

## Data Availability

The data used to support the findings of this study are available from the corresponding author upon request.

## Supplementary Materials

Supplementary Materials DEGs between the sensitive and resistant groups. GO and KEGG pathway enrichment analyses. Protein-protein interaction (PPI) network integration.
