# Supplementary material for: Radiosensitivity-Related Genes and Clinical Characteristics of Nasopharyngeal Carcinoma
Source: Biomed Res Int. 2020 Nov 22;2020:1705867. doi: 10.1155/2020/1705867 (PMC7704138; doi:10.1155/2020/1705867)
Supplement: Supplementary Materials — DEGs between the sensitive and resistant groups. GO and KEGG pathway enrichment analyses. Protein-protein interaction (PPI) network integration. [file 1705867.f1.doc]

**Supplementary**

DEGs between the sensitive and resistant groups

DEGs between two groups were identified using the linear models for microarray data (limma) package in R(genetics and biology, 2004). For our purpose, a P-value cut-off of 0.05 was used for significantly altered expression of genes between two groups. Cluster analysis was used to determine the distribution of these DEGs between two groups. Principal coordinate analysis (PCoA) was employed to identify the expression patterns of these genes between two groups. In addition, the statistical test ADONIS in the R package was also employed to evaluate the expression variation between two groups. The nonparametric multivariate variance test can effectively distinguish between groups based on distance matrices.

GO and KEGG pathway enrichment analyses

The functional and pathway enrichments of the proteins encoded by candidate genes were analyzed, and the genes were annotated using the DAVID database(BT et al., 2007) (https://david.ncifcrf.gov/). Gene Ontology (GO) annotations were performed using a DAVID online tool on the screened DEGs. KEGG pathway analysis of DEGs was performed using the KOBAS 3.0 online analysis database (available online: http://kobas.cbi.pku.edu.cn/). A *P*-value of <0.05 was considered statistically significant.

Protein-protein interaction (PPI) network integration

The STRING database (http://string-db.org/) is a software system that is commonly used to identify the interactions between known proteins and predicted proteins. The core of the Cytoscape software is a network. Each node is a gene, protein, or molecule, and the connections between nodes represent the interaction of these biological molecules, which can be used to identify interactions and pathway relationships between the proteins encoded by DEGs. The corresponding proteins in the central node may be core proteins or key candidate genes with important physiological regulatory functions.

**References**

BT, S., W, H.d., Q, T., Y, G., S, B., D, L., et al. (2007). DAVID Knowledgebase: a gene-centered database integrating heterogeneous gene annotation resources to facilitate high-throughput gene functional analysis. 8(undefined)**,** 426.

genetics, S.G.J.S.a.i., and biology, m. (2004). Linear models and empirical bayes methods for assessing differential expression in microarray experiments. 3**,** Article3. doi: 10.2202/1544-6115.1027.
